# SARS-CoV-2 infection and psychological distress : a prospective sero-survey in southern Switzerland

**DOI:** 10.1192/j.eurpsy.2024.337

**Published:** 2024-08-27

**Authors:** B. Bano, C. Sculco, G. Piumatti, R. Amati, M. Purgato, E. Albanese

**Affiliations:** ^1^ Institute of Public Health, Università della Svizzera italiana; ^2^Institute of Public Health, Universita’ della Svizzera italiana, Lugano, Switzerland; ^3^Fondazione Agnelli, Turin; ^4^Department of Neurosciences, Biomedicine, Movement Sciences, University of Verona, Verona, Italy

## Abstract

**Introduction:**

The COVID-19 pandemic has had an impact on the mental health of the population that, to some extent, may be due to the neurotropism of SARS-CoV-2. However, evidence is extremely sparse on the prospective association between serological evidence of COVID-19 infection and psychological distress.

**Objectives:**

We aimed to explore the prospective association between seropositivity and psychological distress – assessed by symptoms of depression, anxiety and stress – in the general adult population in southern Switzerland. Further, we investigated whether this association varied over time and between pandemic waves.

**Methods:**

We used data from 305 adults who participated in the Corona Immunitas Ticino (CIT) prospective sero-survey cohort study. We tested the association between serologically confirmed SARS-COV-2 infection at baseline (June–December 2020) and depression, anxiety and stress scores as measured by the DASS-21 scale at three time points between December 2020 and March 2021, also taking into account for sociodemographic characteristics (age, gender, education level, presence of chronic diseases, smoking, obesity).

**Results:**

In our sample, 84.3% (mean age of 51.30, SD= ± .93) were never infected. Seropositive participants were significantly younger on average (M=46.90, SD= ±2.00, P= .04). At the first follow-up (see Table 1), seropositive participants had higher rates of mild conditions for depression (OR= .64; P= .014) and anxiety (OR= .50; P= .030), than seronegatives. Overall, after the 6-month follow-up, seropositive participants had significantly lower rates of mild conditions for DASS-21 subscales. In addition, prevalence of mild conditions for depression, anxiety and stress decreased more rapidly over time among infected vs. never infected (see Figure 1). Older age and the presence of chronic diseases were associated with mild anxiety (OR= .97; P= .013; OR=3.47; P= .001) and stress (OR= .96; P= .003; OR= 2.56; P= .010).

**Table 1.** Associations (Odds Ratios) between seropositive immunological status and mental health between December 2020 and March 2021 in Ticino, southern Switzerland (N=305)

DASS-21 defined mild condition	OR	P value	CI (95%)
Depression	0.641	0.014	0.449 – 0.914
Anxiety	0.502	0.030	0.270 – 0.936
Stress	0.712	0.113	0.468 – 1.083

Note. Generalized estimating equation models results. Co-variates include time, age, gender, chronic diseases, obesity, smoking and education level.

**Conclusions:**

Our results provide new evidence on the association between COVID-19 seropositivity and poor mental health and underline the public health implications of the pandemic because the number of infected individuals largely exceed the 770 million of recorded COVID-19 (symptomatic) cases.

**Disclosure of Interest:**

None Declared

